# Distinct Risk Profiles for Human Infections with the Influenza A(H7N9) Virus among Rural and Urban Residents: Zhejiang Province, China, 2013

**DOI:** 10.1371/journal.pone.0095015

**Published:** 2014-05-02

**Authors:** Fan He, Meng Zhang, Xinyi Wang, Haocheng Wu, Xiaopeng Shang, Fudong Li, Chen Wu, Junfen Lin, Bao-Ping Zhu

**Affiliations:** 1 Zhejiang Provincial Center for Disease Control and Prevention, Hangzhou, People's Republic of China; 2 Chinese Field Epidemiology Training Program, Chinese Center for Disease Control and Prevention, Beijing, People's Republic of China; 3 Dongguan Municipal Center for Disease Control and Prevention, Dongguan City, People's Republic of China; The University of Tokyo, Japan

## Abstract

**Objective:**

To identify the risk factors and source of infection leading to human infections with the Influenza A(H7N9) virus in urban and rural areas.

**Methods:**

We conducted a case-control investigation to identify potential exposures and risk factors. Controls were randomly selected from the same community as the cases using random digit dialing. We used exact conditional logistic regression to evaluate the exposures and risk factors, stratified by urban and rural residence.

**Results:**

Buying live or freshly slaughtered poultry from a market was significantly associated with illness onset among both urban [48% of 25 case-patients and 12% of 125 control-persons, adjusted odds ratio (AOR) = 19, 95% CI: 2.3–929] and rural (33% of 18 case-patients and 8.9% of 90 control-persons, AOR = 13, 95% CI:1.5–∞) residents. In rural area, tending to home-raised poultry (56% of 18 case-patients and 10% of 90 control-persons, AOR = 57, 95% CI: 7.5–∞) and existence of a poultry farm in the vicinity of the residence (28% of 18 case-patients and 5.6% of 90 control-persons, AOR = 37, 95% CI: 3.8–∞) were also significantly associated with disease onset. Presence of underlying medical conditions was a significant risk factor for urban residents (76% of 25 case-patients and 13% of 125 control-persons, AOR = 49, 95% CI: 7.1–2132).

**Conclusions:**

Buying live or freshly slaughtered poultry from a market is a risk factor for both urban and rural residents, tending to home-raised poultry and existence of a poultry farm in the vicinity of the residence are risk factors unique for rural residents. The virus might have been in stealth circulation in the poultry population before infecting humans. We recommend strict poultry market management and multisectoral collaboration to identify the extent of poultry infection in China.

## Introduction

Since the outbreak of severe human infections with the novel reassortant influenza A (H7N9) virus (H7N9 virus) was identified in March 2013 [1], 132 confirmed cases of human infection with the H7N9 virus (including 43 deaths) have been reported in mainland China as of 31 July 2013 [2]–[4]. Of those 132 confirmed case-patients, 44 (including 10 deaths) were residents of Zhejiang Province in southeastern China (2011 total population 55 million; urban 34 million, rural 21 million), representing the highest case count of all provinces.

Although much has been learned about the virological characteristics of this novel virus [5]–[7], many knowledge gaps still exist, especially regarding the epidemiology of human infections [8]–[9]. Among the critical and pressing questions are, what the natural reservoir for the virus is, whether the virus was circulating in the local poultry farms or transported from elsewhere before causing the human outbreak, and why older men were disproportionally affected during this outbreak [3].

We conducted an investigation to identify the risk factors and source of infection leading to human infections with the H7N9 virus, stratified by rural and urban residency in Zhejiang province, to shed some light on whether the H7N9 virus iss present in local poultry at the time humans infected, and to understand the distinctive age- and sex- distribution of the case-patients.

## Materials and Methods

### Participants

Urban area in this paper includes cities, towns and suburbs. Rural area refers to countryside. Based on the Guidelines for the Diagnosis and Treatment of Human Infections with the Influenza A (H7N9) Virus issued by the National Health and Family Planning Commission of the People's Republic of China [10], we defined a confirmed case of the H7N9 virus infection as clinical symptoms consistent with acute influenza (fever, cough, coryza, difficulty breathing) or with a history of contact with a confirmed or suspected case and a laboratory test positive for avian influenza A (H7N9) virus; PCR, viral isolation or a four-fold or greater increase in serum antibodies specific for this virus isolated in paired sera.

We selected controls based on the following matching criteria: same sex, age±5 years, free of respiratory symptoms (e.g., cough, sore throat, temperature ≥38°C) within two weeks of illness onset of the matched case-patient. We selected controls randomly in the same community or village of the matched case-patient by the Mitofsky-Waksberg random digit dialing method [11]. Once a telephone call was answered, we asked if there was a household member meeting the criteria. We would continue to call the next family if no appropriate control found in the current call. For a family which has a member matching our criteria, we conducted a detailed investigation on that member. In addition we collected information of demography, live poultry exposure and live poultry market exposure for the rest household members. We asked information relating to matching criteria for all household members, and identified one most closely meeting the matching criteria as the control-person. In this manner, we selected five controls for each case.

### Data collection

We developed a data collection questionnaire to record the information for case and control-persons, regarding demographic characteristics, underlying medical conditions [mainly chronic diseases such as asthma, chronic bronchitis, pulmonary tuberculosis and other chronic lung diseases, diabetes, hypertension, cancer, heart disease, kidney disease, chronic liver disease, immunodeficiency and so on], tobacco use, and history of potential exposures [including visiting live poultry market, buying live or freshly slaughtered poultry, raising poultry at home or around the house, tending to poultry (e.g., tending to sick birds, cleaning hen-houses, feeding poultry, etc.), existence of a poultry farm in vicinity of residence (radius of three thousand meters), having neighbors that raised poultry at home] We conducted in-person interviews for the case-patients or, in the case of critical or deceased case-patients, proxy interviews of the patients' family members. We collected the exposure information during the 10 days prior to illness onset for the case-patients, and a comparable 10-day period for the control-patients. Case-patients were interviewed during April 13-May 2, 2013, whereas due to the lack of human resources during the acute phase of the outbreak response, interviews for control-persons did not occur until June 15–20, 2013.

### Statistical analysis

We used the univariate exact conditional logistic regression analysis [12] to compare exposure histories and risk factors between case- and control-persons. To control for confounding, we conducted multivariate exact conditional logistic regression analysis of exposure variables separately for urban and rural residents. Statistical analyses were performed by using SAS (version 9.13). Variables with p<0.05 were included in the final models.

### Laboratory testing

All cases of H7N9 virus infection were confirmed by rRT-PCR or viral isolation, following standard laboratory protocols [10].

### Ethical review

This investigation was part of a response to a public health emergency; therefore, ethical review was exempt by the Ethics Committee at the Zhejiang Provincial CDC. Because most patients were in serious condition and all control-persons were interviewed by telephone, we had applied to the Ethics Committee for verbal informed consent before starting the interviews instead of written consent. We developed a linelist (including name, telephone, respondence and reason of rejection, etc.) to record participant consent.

## Results

**Figure 1:**
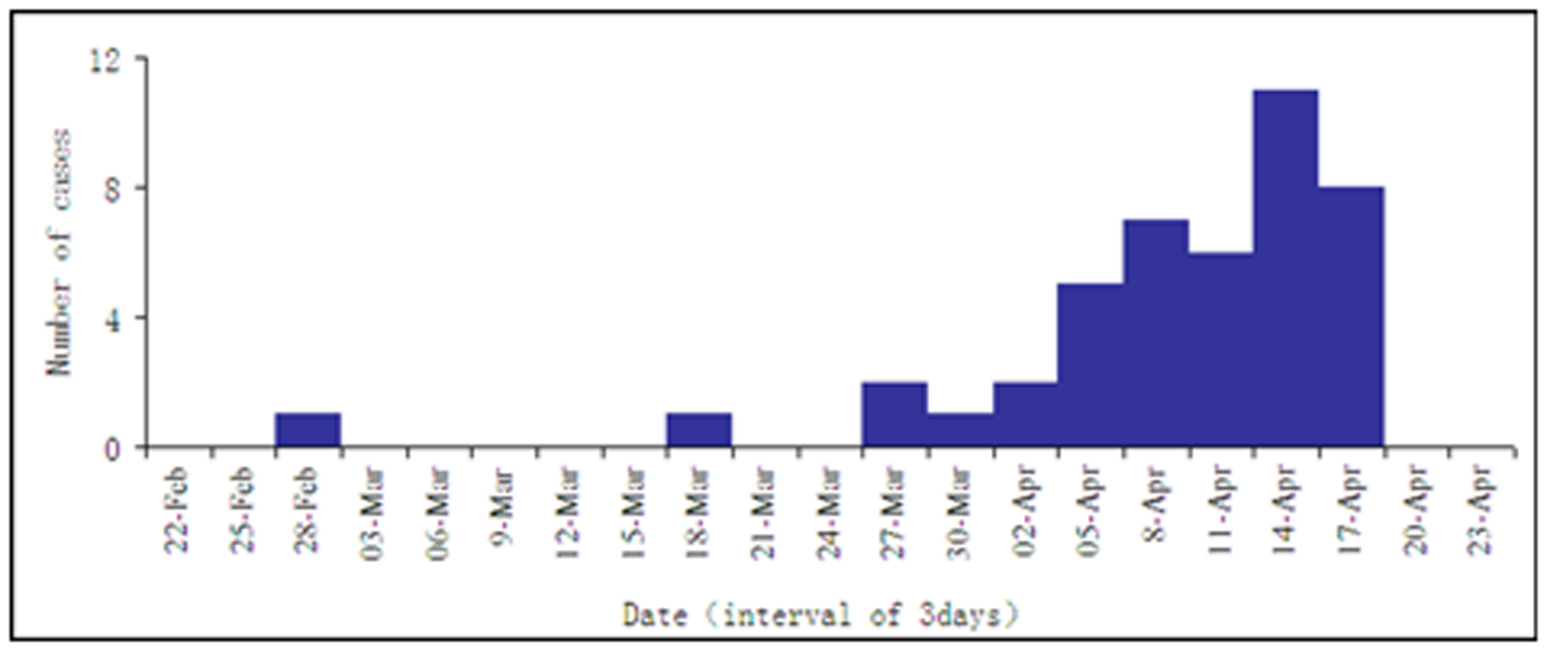
Onset time of 44 confirmed cases of influenza A (H7N9) virus human infection — Zhejiang Province, China, 2013.

**Figure 2:**
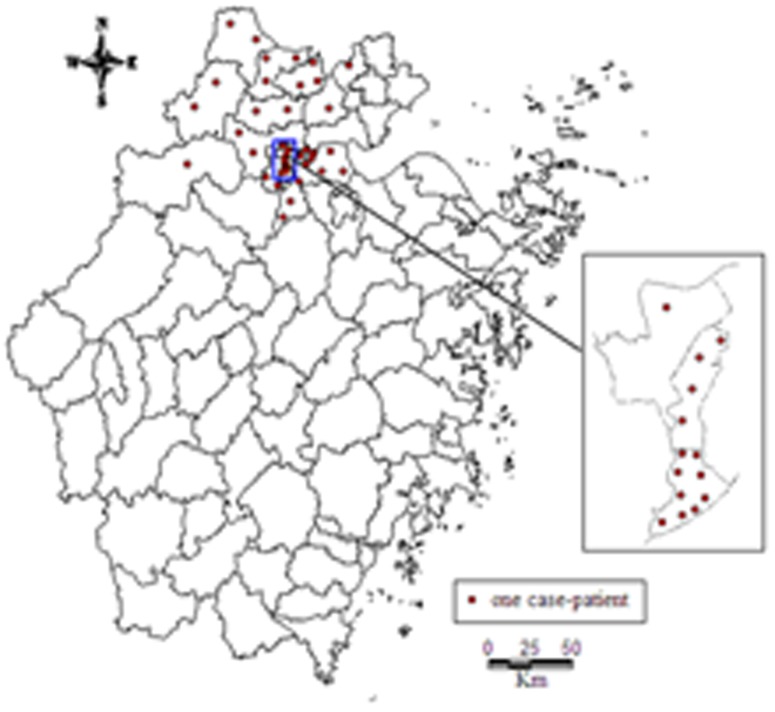
Geographic distribution of 44 confirmed cases of influenza A(H7N9) virus human infection — Zhejiang Province, China, 2013.

Of the 44 confirmed case-patients in Zhejiang province, 25 resided in the urban area of the province, while 19 resided in rural area. The onset dates of the case-patients were between March 7 and April 18, 2013 (Fig. 1). Case-patients were mostly elder men (men = 63%; median age = 63 years; age range = 31–86 years). All 44 cases occurred in the 15 northern districts and counties, out of a total of 90, in Zhejiang Province (Fig. 2).

Of all 44 confirmed case-patients, 43 consented to the interview. For these case-patients, we identified 215 control-persons matched by age-, sex-, and place of residence. Case-patients and control-persons did not differ significantly by age, sex, place of residence, educational level, and occupation (Table 1).

**Table 1:** Characteristics of case- and control-persons in the study of risk factors for influenza A (H7N9) virus human infections — Zhejiang Province, China, 2013.

Characteristics	case(n = 43)	%	control(n = 215)	%	P
Age (years), median (range)	63(31–86)		61(28–84)		0.98
28–29	0	0	2	1	
30–59	20	47	95	44	
60–86	23	53	118	55	
Sex					1.0
Men	27	63	135	63	
Women	16	37	80	37	
Place of residence					1.0
Urban area	25	58	125	58	
Rural area	18	42	90	42	
Education level					0.98
Primary school or below	17	40	78	36	
Junior middle school	16	37	80	37	
Senior middle school	7	16	37	17	
College or higher	3	7.0	20	9	
Occupation					0.75
Self-employed	6	14	31	14	
Full-time employees	8	19	42	20	
Home-maker	0	0	7	3.0	
Farmer	12	28	67	31	
Retiree	14	33	61	28	
Unemployed	3	7.0	7	3.0	

In the univariate analysis stratified by place of residence, buying live or freshly slaughtered poultry and having neighbors that raised poultry at home were common exposure factors significantly associated with disease onset in both urban and rural areas. In the rural area, raising poultry at home or around the house, tending to home-raised poultry (e.g., feeding the birds, caring for sick birds, and cleaning the henhouses), and existence of a poultry farm in the vicinity of the residence were additional exposure factors significantly associated with disease onset (Table 2). Presence of underlying medical conditions (such as COPD, cardiovascular diseases, hypertension, and diabetes) was significantly associated with disease onset for both urban and rural residents; on the other hand, smoking was significantly associated with disease onset only among urban residents (Table 2).

**Table 2:** Univariate exact conditional logistic regression analysis of potential exposures and risk factors for influenza A (H7N9) virus human infection — Zhejiang Province, China, 2013.

Exposures/risk factors*	Overall				Urban				Rural			
	% exposed		OR	95% CI	% exposed		OR	95% CI	% exposed		OR	95%CI
	Case (n = 43)	Control (n = 215)			Case (n = 25)	Control (n = 125)			Case (n = 18)	Control (n = 90)		
Buying live or freshly slaughtered poultry	42	11	7.9	3.0–23	48	12	8.9	2.6–39	33	8.9	6.6	1.3–42
Raising poultry at home or around the house	33	10	9.0	2.6–39	12	2.4	6.4	0.70–78	61	21	11	2.3–111
Tending to poultry	26	4.7	19	4.0–182	4.0	0.80	5.0	0.06–392	56	10	33	4.4–435
Existence of a poultry farm in vicinity of residence	16	2.8	13	2.4–133	8.0	0.80	10	0.52–590	28	5.6	16	1.6–788
Having neighbors that raised poultry at home	56	20	6.4	2.9–15	44	13	5.1	1.8–15	72	30	9.8	2.4–59
Presence of underlying medical conditions	65	22	7.1	3.2–17	76	13	30	7.0–272	50	18	4.7	1.4–19
Chronic obstructive pulmonary disease	24	0	31	4.2–∞	12	0	18	1.9–∞	11	0	11	0.84–∞
Cardiovascular disease	30	0	41	6.1–∞	16	0	21	2.6–∞	17	0	18	1.9–∞
Hypertension	54	13	8.1	3.1–24	56	11	23	5.1–215	28	16	2.6	0.60–12
Diabetes	33	3.7	5.7	1.6–23	24	2.4	11	1.9–117	11	4.4	2.1	0.17–20
Other diseases	39	0.55	61	9.8–∞	32	0	50	7.7–∞	11	1.1	9.7	0.75–∞
Smoking cigarettes	44	25	2.9	1.3–6.6	48	19	5.0	1.6–17	39	32	1.4	0.37–5.2

*The following exposures and factors were evaluated but were not statistically associated with disease onset: Slaughtering poultry, contact with dead poultry, visiting lake/park/pond/paddy field.

When we conducted separate multivariate exact logistic regression analysis by place of residence, buying live or freshly slaughtered poultry from a live poultry market was a common significant exposure factors in residents of both urban [adjusted odds ratio (AOR) = 19, 95% confidence interval (CI): 2.3–929] and rural areas (AOR = 13, 95% CI: 1.5-∞). In the rural areas, tending to home-raised poultry (AOR = 57, 95% CI: 7.5-∞) and presence of a poultry farm in the vicinity of the residence (AOR = 37, 95% CI: 3.8-∞) were additional significant exposure factors. Existence of underlying medical conditions was a significant risk factor among urban residents, but not among rural residents (Table 3).

**Table 3:** Exact conditional logistic regression analysis of exposures and risk factors for influenza A(H7N9) virus human infection, by urban or rural residence — Zhejiang Province, China, March – 2013.

Exposures/risk factors	Overall		Urban		Rural	
	Adjusted Odds Ratio	95% Confidence Interval	Adjusted Odds Ratio	95% Confidence Interval	Adjusted Odds Ratio	95% Confidence Interval
Purchase of live or freshly slaughtered poultry	4.9	1.2–24	19	2.3–929	13	1.5–∞
Tending to home-raised poultry	9.9	0.40–318	NS#		57	7.5–∞
Existence of poultry farm in vicinity of residence	42	2.3–1000	NS#		37	3.8–∞
Presence of underlying medical conditions	6.7	2.3–23	49	7.1–2132	NS#	

# NS = Association not statistically significant (p>0.05).

To understand why elder men over-represented in case-patients during this outbreak, we analyzed the data of exposure history to live poultry market during the outbreak period among the control-persons and their household members, by sex and place of residence. For this analysis, we selected control-persons and their household members between the ages of 31–86 years, i.e., the same age range as the case-patients. We found that among the control-persons and their household members, men were 2.6 times as likely as women to buy live or freshly slaughtered poultry from a poultry market in the urban area; on the other hand, in the rural area, this ratio was 0.89∶1(table 4). These sex ratios were consistent with those in case counts in urban (3.2∶1) and rural (0.80∶1) areas in Zhejiang Province.

**Table 4:** Exposure to the live or freshly slaughtered poultry from a poultry market of control-persons and their household members.

Age	Overall				Urban				Rural			
	Male (N = 182)		Female (N = 240)		Male (N = 109)		Female (N = 136)		Male (N = 73)		Female (N = 104)	
	n	%	n	%	n	%	n	%	n	%	n	%
28–29	5	4.7	6	7.5	1	1.3	3	10	4	12	3	5.9
30–59	66	62	45	56	48	64	15	52	18	56	30	59
60–86	36	34	29	36	26	35	11	38	10	31	18	35
**Total**	107	100	80	100	75	100	29	100	32	100	51	100

## Discussion

In this case-control study, we explored differences in risk profiles for the H7N9 virus human infection between urban and rural residents in Zhejiang province. Our investigation showed that, while buying live or freshly slaughtered poultry from a market was a common significant exposure factor for both urban and rural residents, tending to home-raised poultry and existence of a poultry farm in the vicinity of the residence were significant exposure factors unique for rural residents. Our result is basically in accordance with that in a case-control study in Jiangsu province [13]. However, we found raising poultry at home or around the house a risk factor while they didn't.

### Exposures to Live Poultry Market

Exposures in live poultry market are known to be associated with human infections with the H5N1 avian influenza virus as well as with the H7N9 virus [13]–[15]. Live poultry markets are usually heavily contaminated by poultry secreta, feces and viscera. During the current outbreak of human infections with the H7N9 virus, environmental samples (including poultry faeces, garbage and sewage) collected from the live poultry markets that the case-patients frequently visited were repeatedly tested positive for the H7N9 virus [16]–[17]. Hence visiting these markets exposes the visitors to risk of H7N9 virus infection. In April of 2013, soon after the outbreak of human infections with the H7N9 virus was identified, the Zhejiang provincial government ordered all live poultry markets throughout the province to be closed. Subsequently, human infection cases sharply decreased [16], which provided additional credence that live poultry markets might have played an important role in facilitating human infections with the H7N9 virus.

### Exposures to Tending to Poultry and Existence of Poultry Farms

Our investigation revealed that tending to home-raised poultry and existence of poultry farms in the vicinity of the residence were significantly associated with H7N9 virus infection in the rural area. A previous investigation revealed that the first case of the H7N9 virus human infection in Zhejiang province was a rural resident who did not have a travel history during the 10 days prior to her illness onset [18]. In addition, a recently published serological study conducted in Zhejiang province showed that 6.3% of poultry workers had antibody to the H7N9 virus [19]. Also, an anecdotal internet report in April 2013 indicated that the H7N9 virus had been detected on a poultry farm in Zhejiang province [20]. These findings, taken as a whole, indicate that the H7N9 virus causing human infections in Zhejiang province might have come from infected local poultry, rather than from poultry imported from other provinces. During the earliest stage of the outbreak, the first cases of human infection occurred in Shanghai municipality and Zhejiang, Anhui, and Jiangsu provinces [4], all closely located in an area in southeast China with high poultry density [21]. Presumably, the reassortment that created the novel H7N9 virus occurred on one of the poultry farms in this area. Because this reassortant H7N9 virus has low pathogenicity to poultry and usually does not cause disease or death in birds [22],hence the virus might have already stealthily spread among the farms via poultry trade. The sudden increase in the demand for live poultry in the period of the traditional Qingming Festival on March 22, 2013, the most important day for tomb sweeping and animal sacrifices to ancestors (usually with chicken) in the Chinese culture, might cause a sharp increase in human contact with the infected poultry, leading to the outbreak of human disease [18]. The subsequent culling of all poultry ordered by the governments of Zhejiang and other provinces in southeastern China might effectively halt the outbreak.

### Underlying Medical Conditions

As with other published studies [13], [23], our investigation showed that underlying medical conditions were associated with onset of human infection with the H7N9 virus. However, the question on whether underlying medical conditions are risk factors for infection with the H7N9 virus *per se*, or they are risk factors for developing pneumonia after infection with the virus, remains to be elucidated.

### High Men-to-Women Ratio

One of the mysteries during this outbreak was the over-representation of elder men in the case-patients. Our findings showed that the high men-to-women ratio in the case count only existed in the urban area. Further, our data for the control-persons and their household members (which roughly represented the general population) suggested that the disproportionally high case count in urban men was due to their higher exposure opportunities to the live poultry market. However, we cannot rule out the effect of underlying chronic diseases on elder patients, which might decrease immune function [13], [24]–[25].

Several important questions regarding this new influenza virus remain unanswered. These include: Is there a natural reservoir for this virus in the wild bird population? How widespread is the virus in the poultry population in China? How is the virus spread among the poultry from one area to another? To answer these questions, collaborative research efforts involving public health, agriculture, and other relevant sectors in China are critically important.

### Limitations

Our study had three major limitations. First, we conducted the interviews of the case-patients during April–May 2013, immediately after their disease diagnosis, whereas due to resource limitations during the acute phase of the outbreak we did not interview the control-persons until June 15–20. This delay in the interviews of control-persons might result in recall bias and information bias. Recall bias could cause the effect estimates to go in either direction. On the other hand, because the public was more aware of the risk of live poultry exposures and other risk factors in June than in April-May, the control-persons might report more risk factors in June than they would have in April–May, thus resulting in conservative effect estimates. Second, we collected the data from case-patients by face-to-face or proxy interviews, whereas the control-persons were interviewed by telephone, potentially resulting in information bias. Third, we did not perform serological tests for control-persons and might underestimate the role of risk factor. However, serological test for close contacts of case-patients revealed a very low positive rate. We will further carry out seroepidemiology study among population in the future.

## Conclusions

In summary, we found that while exposure to the live or freshly slaughtered poultry was significantly associated with the H7N9 virus human infection, exposures to poultry raised at home or on a nearby poultry farm were also significantly associated with increased risk in the rural area of Zhejiang province. We recommend implementation of strategies similar to those adopted by the Hong Kong authorities in controlling the H5N1 virus outbreak, such as regular “rest days” of live poultry market, banning of overnight poultry storage, and slaughter of poultry in a designated area of live poultry market [26]. In addition, efforts should be made to persuade residents (especially rural residents) not to raise poultry inside or around their houses [27].
